# DEVELOPING A RISK ASSESSMENT AND BRIEF INTERVENTION FOR ELDER MISTREATMENT: A MIXED-METHODS STUDY

**DOI:** 10.1093/geroni/igae098.2052

**Published:** 2024-12-31

**Authors:** Monique Pappadis, Leila Wood, Karen Schlag

**Affiliations:** The University of Texas Medical Branch, Galveston, Texas, United States; The University of Texas Health Science Center, Houston, Texas, United States; The University of Texas Medical Branch, Galveston, Texas, United States

## Abstract

Elder mistreatment (EM) is public health and safety concern for older adults, especially those with mild cognitive impairment and Alzheimer’s Disease and Related Dementias (MCI/ADRD); however, few tools exist to assess risk or address risks, especially those that may come from caregivers or the social environment. To address these gaps, we used Medicare outpatient data (2015-2018) and semi-structured interviews with providers, older people with MCI/ADRD, and family caregivers to: 1) Identify risk and protective factors for EM, 2) Understand education and training gaps that may contribute to risk, and 3) Develop a screening, brief intervention, and referral to treatment (SBIRT) model for caregivers and older adults with MCI/ADRD to address risks for EM. Our preliminary findings indicate that social determinants of health, such as housing, financial instability, and social isolation, along with frailty, contribute to EM risk. Caregiver stress, often from financial instability, can increase risk conditions for EM. The lack of caregiver and provider education on EM contributes to a lack of identification of risk factors. Thus, the developed SBIRT model centers on risk assessment, universal education, and referral to support based on the results of the risk assessment. Preliminary feasibility and acceptability of the SBIRT intervention will be reviewed, along with a review of risk assessment protocols. The session will end with describing the next steps to expanding the SBIRT intervention to a greater number of primary care clinics across a large academic health science center’s system.

